# Utilization of free maternity services among women of child bearing age in Machakos County, Kenya

**DOI:** 10.11604/pamj.2021.39.25.26499

**Published:** 2021-05-10

**Authors:** Alice Mukunzu Ngesa, Joyce Kirui, Isaac Matheka, George Otieno, Alison Yoos

**Affiliations:** 1 School of Public Health and Applied Human Sciences, Kenyatta University, Nairobi, Kenya,; 2 Department of Health Management and Informatics, Kenyatta University, Nairobi, Kenya,; 3 Department of Health and Emergency Services, Machakos County, Kenya,; 4 Improving Public Health for Action (IMPACT) training program, Nairobi, Kenya

**Keywords:** Free maternity services, utilization of free maternity services, post natal women

## Abstract

**Introduction:**

globally, the rate of maternal mortality is unacceptably high with Kenya recording a rate of 362 maternal death per 100,000 live births. Even so, only 62% of women deliver under skilled health care. The government of Kenya introduced Free Maternity Services (FMS) to all women delivering in public health facilities as a way of increasing facility-based deliveries. Despite this, intervention, health facility deliveries in Machakos County are still low. This study aimed to identify hindrances and enablers of the FMS program in Machakos County.

**Methods:**

it was a cross-sectional study conducted among postnatal women who delivered between September 2018 and September 2019 in Machakos County. A total of 394 women were enrolled. Data was collected using questionnaires and focus group discussions. Key informant interviews were conducted using nursing officer in charge of selected health facilities. Qualitative data was analyzed using chi-square and fishers exact. Multivariate logistic regression was used to determine predictors of utilization of FMS. Statistical significance was set at p < 0.05.

**Results:**

utilization of FMS in Machakos County was 75.6%. Factors that were associated with utilization of FMS included marital status (p = 0.006), parity (p = 0.038), distance from health facility (p = 0.000), services offered during labour (p = 0.000), treatment of mothers by healthcare workers during labour (p = 0.000), provision of adequate food (p = 0.005), quality of service (p = 0.000) and cleanliness of the maternity ward (p = 0.000).

**Conclusion:**

utilization of FMS in Machakos County is optimal. Health facilities should be supported to offer FMS by providing them with necessary supplies.

## Introduction

Globally, approximately 830 women die each day from preventable pregnancy and childbirth related complications, 99 per cent of which occur in developing countries [1]. Global statistics indicated large disparities in the maternal mortality ratio (MMR) between low income and high-income countries of 239 per 100,000 live births and 12 per 100,000 live births respectively [2]. These disparities also occur within a country as seen between women with high and low income and between women living in rural versus urban areas [1]. Even so, sub-Saharan Africa and South Asia account for 88 per cent of global maternal deaths [3] with sub-Saharan Africans contributing the highest MMR of 546 maternal deaths per 100,000 live births [3], this being 66 per cent of all global maternal deaths per year. In Kenya, MMR has declined from 488 maternal deaths per 100,000 live births in 2008 to 362 maternal deaths per 100,000 live births currently [4].

Non utilization of skilled birth attendants during delivery has been cited as a contributor to the high MMR in resource limited settings [5]. As such, health facility delivery services have been promoted as one of the solutions to preventing maternal and neonatal deaths [6]. However, the cost of care has been cited as one of the barriers to utilizing skilled birth attendance services in developing countries [5]. In line with this observation, several African countries have either reduced or eliminated hospital delivery fees as a way of promoting utilization of health facility delivery [7]. Consequently, Kenya abolished payment of delivery fees in all public health facilities on June 1, 2013 and instead replaced it with the free maternity service (FMS), a policy meant to promote utilization of health facility-based deliveries by reducing the financial burden and high costs of maternity services [8].

Despite the several efforts that the Kenyan government has put in place aimed at reducing maternal mortality ratio for example free maternity services (FMS) program and scrapping of user fees in level two and three health facilities, the MMR is still high (362 per 100,000 live births) while utilization of skilled birth attendants is low [9]. Machakos County health statistics for the years 2013 to 2017 on delivery under skilled birth attendant have remained low at 38.2%, 46.5%, 55.5%, 56.8%, and 47.4% respectively. The Machakos County Integrated Development Plan (CIDP) report for 2017 [10] further indicate only 67.2% of pregnant women in the country attended at least one antenatal care clinic while only 47.4% of pregnancies within the county were delivered by a skilled birth attendant. This utilization of skilled birth attendance during delivery is below the 90% target set by WHO [10]. It is against this backdrop that this study sought to identify hindrances and enablers of the free maternity program in Machakos County so as to come up with ways of improving utilization of the free maternity program within Machakos County.

## Methods

### Study design

This was a descriptive cross-sectional study done among households in Masinga sub County of Machakos county, Kenya. Study variables included individual client characteristics, mother´s delivery experiences, mother´s level of awareness on FMS and health facility organizational factors while the outcome variable was utilization of FMS. Utilization of FMS was defined as delivering in a public health facility while not paying for the services.

### Study population

The study population comprised of postnatal women who delivered between September 2018 and September 2019 for the quantitative arm while health workers in charge of health facilities and focus group discussions were selected for the qualitative arm. A sample size of 421 respondents was determined using the Fishers formula for a population of more than 10000.

$$\text{n=}\frac{Z^{2}pq}{d^{2}}$$

n= sample size

z = normal deviate usually set at (1.96) which corresponds to the 95% confidence level

p = proportion of skilled deliveries in Machakos County

q = 1-p

d = degree of accuracy = 0.05

Therefore, at 95% confidence level and +-5 percentage precision and population proportion of 50% the sample size will be:

(1.96*1.96) 0.47(1-0.47)/0.05*0.05

n=3.8416*0.47(0.53)/0.0025

n=383 (desired sample in a population greater than 10000)

n = 383 + 10% non-respondents = 383 + 38 = 421

n = 421

10% of subjects were included to cater for non-responses thus 421 questionnaires were administered. However, after questionnaire checking, cleaning and editing, 394 questionnaires were deemed fit for analysis.

### Sampling technique

Multistage sampling technique was employed to select the respondents. Stage 1- Masinga sub county was purposively selected because it recorded the lowest deliveries aided by a skilled birth attendant in Machakos county [10]. This is despite the sub county having the highest number of public health facilities in the county relative to other sub counties. Stage 2- Three wards were randomly selected through balloting from the five wards of Masinga Sub County. Stage 3- Eight community units were purposively selected from the various community units in the three wards. In these community units, a probability proportional to the population was used to determine the number of households required in each ward. Finally, the women to be included in this study were systematically sampled from their households. If a household had two or more women who qualified for the study, the participant was chosen through balloting.

### Inclusion/exclusion criteria

Consenting women aged between 18 and 49 years who were residents of the study area and delivered within one year to the start of the study were included. On the other hand, non-consenting postnatal women, mentally unstable, those who had memory loss or those who were sick thus unable to participate were excluded from the study.

### Research instruments

Semi-structured questionnaires were used in collection of quantitative data from postnatal women while FGDs were used to gather qualitative data. The questionnaires were administered by trained research assistants in English, and translated in Kikamba where appropriate. Additional qualitative data was collected from key informant interview guides through sessions held with the nursing officer in charge of selected health facility.

### Data analysis

Statistical package SPSS version 25 was used in data analysis. Participants´ sociodemographic characteristics were analyzed descriptively while fisher´s exact test was used to compare characteristics between participants who utilized FMS and those who didn´t. Variables that were found to be significantly associated with FMS (p < 0.05) were fit into a multivariate logistic model to determine predictors of FMS utilization.

### Logistical and ethical considerations

Ethical approval to conduct this study was given by the National Council for Science, Technology and Innovation (NACOSTI) (Registration number: NACOSTI/P/19/48). Further clearance was given by Kenyatta University Graduate School, the Machakos Department of Health and the Masinga Sub County Health Management Team. All necessary information was explained to the potential participants assuring them of their confidentiality, anonymity and freedom to withdraw at any time without any negative consequence and their informed consent was obtained. Once understood the participants signed or thumb printed the consent form depending on their literacy level. Codes were used to identify the respondents.

## Results

### Sociodemographic characteristics of respondents

A total of 421 women were enrolled in this study out of whom 394 fully filled their questionnaire. Slightly more than half of participants (55.3%, n = 218) were aged between 20 and 29 years while one in three (32.2%, n = 127) were aged between 30 and 39 years. Majority of them were protestant Christians (52.6%, n = 204) while 47.4% (n = 184) were Catholics Christians. Three in four of respondents were married (74.6%), n = 294) while 25.4% (n = 100) were single. Education wise, approximately half of the participants (48.3%, n = 190) had primary level education while 39.9%, (n=157) had secondary level education. Majority of participants were unemployed (44.4%, n=175) with approximately one in five (23.4%, n=92) of the participants being farmers. Slightly more than half of participants (54.6%, n = 215) earned a monthly income of less than Kshs. 10,000/=, while one in three (n=104, 32.5%) lacked monthly income. Half of the respondents (50.8%, n = 200) had 2 to 3 children with 3.6% (n = 14) having more than five children.

### Utilization of free maternity services

The study found out that of the 320 respondents who delivered in government facilities (75.6%, n = 242) never paid for the maternity services while (24.4%, n = 78) paid for the services, though the payments done were not official as no receipts were issued. Those who delivered in government facilities and never paid for the services defined utilization of free maternity services.

*One key informant interviewee reported-*

*“Ever since the introduction of FMS, the number of deliveries in a month has tripled as more mothers are now coming to deliver at the facility”*.

*One FGD discussant commented that:*

*“We know that we are not supposed to pay for the maternity services, but some health workers ask for money after a mother delivers, “chai ya daktarin”, “kupanguza daktari jasho” and at times, when a mother does not have the cash she is forced to leave behind her identity card until that time when she is able to raise the money, others are not issued with birth notification forms until that point when they bring the money”*.

### Participant characteristics associated with utilization of free maternity services

As summarized in Table 1, a comparison of individual characteristics was made between participants who utilized FMS and those who did not. The characteristics tested included age of respondent, religion, marital status, level of education, main occupation, monthly income and parity. From the comparison, a statistically significant difference was observed between marital status and utilization of FMS with married persons being more likely to utilize FMS (X^2^ = 7.465, df = 1, p = 0.006) similarly those with low parity were more likely to utilize FMS (X^2^ = 8.437, df = 3, p = 0.038). There was no statistically significant difference between the two groups based on the other variables.

**Table 1:** association between participant socio demographic characteristics and utilization of free maternity services (FMS)

Independent variable	Response	Dependent variable				P
Individual characteristics		Non utilization of FMS (N=74)		Utilization of FMS (N=320)		
		n	%	n	%	
Age	<19	10	23.3%	33	76.7%	0.107
	20-29	46	21.1%	172	78.9%	
	30-39	21	16.5%	106	83.5%	
	>40	1	16.7%	5	83.3%	
Religion	Catholic	32	17.4%	152	82.6%	0.183
	Protestant	44	21.6%	160	78.4%	
Marital status	Single	18	18.0%	82	82.0%	X^2^ = 7.465 df = 1 **p=0.006**
	Married	60	20.4%	234	79.6%	
Level of education	None	1	25.0%	3	75.0%	0.076
	Primary	38	20.0%	152	80.0%	
	Secondary	30	19.1%	127	80.9%	
	University	0	0.0%	7	100.0%	
	Diploma/tertiary college	8	26.7%	22	73.3%	
	Others	1	20.0%	4	80.0%	
Main Occupation	Employed/salaried worker	3	12.5%	21	87.5%	0.871
	Farmer	23	25.0%	69	75.0%	
	Business/self employed	19	20.9%	72	79.1%	
	Unemployed	30	17.1%	145	82.9%	
	Others	3	25.0%	9	75.0%	
Monthly income	<10,000 Kshs	52	24.2%	163	75.8%	0.218
	10,000-30,000 Kshs	5	11.1%	40	88.9%	
	> 30,000 Kshs	0	0.0%	3	100.0%	
	None	21	16.0%	110	84.0%	
Parity	1	24	20.9%	91	79.1%	X^2^ = 8.437 df = 3 **p=0.038**
	2-3	41	20.5%	159	79.5%	
	4-5	12	18.5%	53	81.5%	
	>5	1	7.1%	13	92.9%	

P statistical significance (two sided fishers exact)

### Level of awareness of free maternity services

Nearly all participants (99.0%) were aware that FMS were offered in government health facilities. Similarly, 90% of participants understood that delivery services in public health facilities were not charged. The source of information on FMS to mothers included health care staffs (57.0%), community health workers (45.0%), radio/ TV (39.0%), husband and close relatives (4.0%), and local leaders (1.0%).

*In one FGD discussion, a participant commented-*

*“Almost everyone in the community knows that giving birth in all government facilities is free of charge”*.

### Relationship between awareness and utilization of free maternity services

Despite knowledge of FMS, only 82.0%, (n = 315) of respondents utilized the services. Five respondents utilized the service despite lack of knowledge of it. Even so, there was no statistically significant association between awareness of FMS and utilization of the service (p = 0.589) (Table 2). However, there was a significant association between utilization of FMS and respondent proximity to a facility offering the service (p = 0.000).

**Table 2:** influence of awareness on FMS and proximity of health facility on participants utilization of free maternity services (FMS)

Study Variable		Utilized free maternity services		P
		Yes (N=320)	No (N=74)	
		n (%)	n (%)	
**Awareness of free maternity services**				
	No	5 (50.0%)	5(50.0%)	0.589*
	Yes	315(82.0%)	69 (18.0%)	
**Nearest facility offered free maternity services**				
	No	46 (45.6%)	15 (20.3%	X^2^ = 15.989, df = 1 p=0.000
	Yes	174 (54.4%)	59 (79.7%)	

P statistical significance (two sided fishers exact)*one sided fishers exact

### Influence of mother´s delivery experiences on utilization of FMS

As indicated in Table 3, positive experiences during delivery that had statistically significant association with utilization of FMS included client´s reception at the facility and satisfaction with services rendered in labour. As a result of this, most participants were ready to visit the same facility for delivery as well as recommend the facility to a friend or relative. Pinching, slapping and beating of women during delivery was a negative experience associated with utilization of FMS.

**Table 3:** relationship of mother´s delivery experiences on utilization of free maternity services (FMS) (n = 320)

Variable		Utilized free maternity		p
		No (74)	Yes (320)	
**Reception at the maternity**				
	Poor	1 (50%)	1 (50%)	**0.001**
	Fair	19 (46.3%)	22 (53.7%)	
	Good	35 (25.2%)	104 (74.8%)	
	Excellent	23 (16.7%)	115 (83.3%)	
**Time it took to be attended at the maternity**				
	<15mins	44 (23%)	147 (77%)	X^2^ = 3.018, df = 3, p = 0.389
	16-20 mins	12 (20.7%)	46 (79.3%)	
	21-30 mins	9 (26.5%)	25 (73.5%)	
	>30mins	13 (35.1%)	24 (64.9%)	
**I would visit the same facility for delivery if need be**				
	Agree	64 (21.8%)	230 (78.2%)	**0.000**
	Neither agree or disagree	11 (64.7%)	6 (35.3%)	
	Disagree	3 (33.3%)	6 (66.7%)	
**I would recommend the facility for delivery to a friend/ relative**				
	Agree	64 (21.7%)	231 (78.3%)	**0.000**
	Neither agree or disagree	11 (68.8%)	5 (31.3%)	
	Disagree	3 (33.3%)	6 (66.7%)	
**Satisfaction with labour ward services- during labour**				
	Satisfied	54 (19.8%)	219 (80.2%)	X^2^ = 21.992, df = 2, p = 0.000
	Neither satisfied or dissatisfied	17 (54.8%)	14 (45.2%)	
	Dissatisfied	7 (43.8%)	9 (56.3%)	
**Satisfaction with labour ward services- during delivery**				
	Satisfied	57 (20.1%)	226 (79.9%)	**0.000**
	Neither satisfied or dissatisfied	18 (64.3%)	10 (35.7%)	
	Dissatisfied	3 (33.3%)	6 (66.7%)	
**Satisfaction with labour ward services-after delivery**				
	Satisfied	57 (19.7%)	232 (80.3%)	**0.000**
	Neither satisfied or dissatisfied	17 (73.9%)	6 (26.1%)	
	Dissatisfied	4 (50%)	4 (50%)	
**Experienced - Verbal abuse**				
	Yes	3 (33.3%)	6 (66.7%)	0.46
**Experienced - Pinching/slapping/beating**				
	Yes	4 (80%)	1 (20%)	**0.014**
**Experienced - Delivered alone without assistance**				
	Yes	2 (25%)	6 (75%)	0.620*

P statistical significance (two sided fishers exact), * one sided fishers exact

### Organizational factors associated with utilization of free maternity services

Comparison was made between mother who utilized FMS and those who dint on the influence of organization factors at the facility and their decision to utilize FMS. Factors tested included general treatment from health care workers, cleanliness of the maternity ward, availability of bed linen, state of the bathroom and toilet, privacy, respect to clients and provision of information to clients by health workers and essential services. Similarly, the mothers were requested to rate provision of food, warm water, sharing of beds in maternity ward, adequacy of health workers and quality of services at their last facility of delivery. From the data analysis, there was a statistically significant association between these factors and participants decision to utilize FMS (p < 0.05). However, provision of basin, sanitary pads and bating soap were not associated with decision to utilize FMS p = 0.131, p = 0.617, p = 0.094 respectively.

### Determinants of utilization of free maternity services

Logistic regression tests were used to develop multivariate models for predicting utilization of FMS from the independent variables. Variables that were significant under logistic regression were included in the multivariate analysis. As summarized in Table 4, having a monthly income were (OR 1.132, CI 1.001-1.279), provision of adequate food after delivery (OR 0.276, CI 0.078-0.984), providing mothers with a a basin after delivery (OR 3.550, CI 1.072-11.762), and adequacy of health workers in maternity ward (OR 3.011, CI 1.454-6.234) were predictors of a person utilization of FMS.

**Table 4:** multivariate models for predicting utilization of free maternity services (FMS)

Predictor variable		Wald	OR	95% C.I.			P
				Lower	Upper		
**Socio demographics**							
	Monthly income	3.927	1.132	1.001	1.279		0.048
**Organizational factors and utilization of FMS**							
	Adequate food was provided	3.942	0.276	0.078	0.984		0.047
	Adequate health workers in the maternity	8.808	3.011	1.454	6.234		0.003
	I was provided with basin	4.298	3.55	1.072	11.762		0.038
							

## Discussion

At 75.6%, the level of utilization of FMS in Machakos county is higher than the national average though lower than the WHO target of 90% [11]. Individual participant characteristics that were associated with utilization of FMS included participant age with middle-aged utilizing FMS more compared to younger and older women. Babalola and Fatusi [12] in a study in Ethiopia reported women of middle childbearing ages as the ones most likely to use FMS compared to their peers in the early or late childbearing ages [12]. In our study, level of education had no role in determining utilization of FMS even so, the service was mainly utilized by participants with primary level of education. Education has been reported to plays a significant role in demystification of poor delivery outcome related beliefs. Our findings are contrary to those by Beatrice *et al*. [13] who reported most clients with higher educational level demand more information on quality of care provided and try to build trust with physicians [13], Obasi [14] also reported education increases the level of awareness and need to use skilled maternity services among women.

The number of children one had was found to influence utilization of FMS with a reduction in utilization of the service among women with more than three children. Women with high parities with normal vaginal deliveries have been reported to prefer delivering at home than hospital [15]. Our findings are contrary to those of Kwast and Liff [16] who reported increases health service utilization for delivery as the number of pregnancies increased [16]. Tsegay *et al*. [17] also reported women with higher parity and successful vaginal deliveries as having more confidence and less fear for pain or risky pregnancy outcomes. Our study found FMS to be mainly utilized by unemployed. Similar findings have been reported by Christine MK [18] and Nahar S [19] who found that housewives/non-working women as being likely to use free maternal health services compared to their employed counterparts. Level of income has also been reported to influence use of FMS with low-income earners [18] being the main ones who utilize FMS. There were high levels of awareness of FMS (99%) among study participants with persons living near a government facility being likely to utilize FMS during delivery. None the less, there was no association between awareness of FMS and utilization of the service. These findings are contrary to those of a study conducted in Australia [20], which found a strong positive correlation between knowledge on maternal issues and utilization of maternal health care.

Most of the tested indicators of mother´s delivery experience were found to influence utilization of the FMS. Services that were rated good or excellent were found to create friendliness which enables women to create good rapport and establish trust with clinician´s thus higher perceived quality of care. Ochako *et al*. [21] and Margaret *et al*. [22] found when mothers perceive care providers to being unsympathetic and having poor attitudes towards women in labour, mistrust is created between them thus reducing their satisfaction levels hence affecting the utilization of the services. As a result of satisfaction with services offered, majority of the women in our study reported they would both consider delivering in the same facility as well as recommending it to their friends. Sallam *et al*. [23] reported mother´s attitude towards healthcare is determined by their experience with the healthcare or what they observe others going through in maternal care. The positive relationship, between attitude and utilization of maternal health care was also experienced in Japan [24].

Facility cleanliness had significant influence on maternal satisfaction with perceived facility cleanliness being associated with high quality service provision. Butler *et al*. [25] argued that the physical birthing environment in most cases, affects patient safety and health, effectiveness of care and the morale of the care providers reported similar results. This was further supported by a report by the World Health Organization, which explained that delivery in unhygienic conditions without the assistance of a skilled birth attendant might result in adverse health conditions of pregnant women consequently reducing their satisfaction levels hence utilization [26]. The current study revealed that majority of women who utilized free maternity services reported that the health workers ensured their privacy. Utilization of maternity services increased with provision of privacy. This is because patients feel valued as the health care workers respects their rights to dignity, privacy and confidentiality. The results concur with a study done by Ochieng [27] who argued that provision of patient privacy affects the health seeking behaviours and ultimately the effectiveness of such care. Provision of patient privacy encourages more women to use the available maternal services since they feel satisfied with service delivery components at their disposal. The results also concur with a study done by Okoth [28] which revealed that there was a relationship between privacy in service delivery and utilization of maternal and neonatal health care services.

## Conclusion

The study found that monthly income, provision of basin, provision of adequate food and adequacy of health care workers were predictors for utilization of FMS. Utilization of FMS in Machakos County public hospitals was optimal though mothers who utilized free maternity services were still reporting illegal payments. Individual client characteristics were not significantly associated with utilization of FMS. The facility staff played a greater role in creating awareness on FMS, however, mothers´ level of awareness was not significantly associated with utilization of FMS. The study concludes that mothers´ delivery experiences played a significant role towards utilization of FMS. The findings conclude that organizational factors played a significant role towards utilization of FMS.

### What is known about this topic

- Utilization of free maternity services increased significantly since the roll out of the free maternity policy in Kenya;
- Despite the maternity services being free in Kenya mothers´ still pay for the services.

### What this study adds

- Factors influencing utilization of free maternity services in Machakos County include parity, marital status, monthly income, proximity to health facility and quality of health care services;
- This study has shown that awareness on free maternity services is not associated with utilization of free maternity services in Machakos County.
